# Simultaneous Myasthenic Crisis With Thyrotoxic Crisis in an Adult Male: An Autoimmune Overlap

**DOI:** 10.7759/cureus.44366

**Published:** 2023-08-30

**Authors:** Nishtha Manuja, Sunil Kumar, Sourya Acharya, Varun Daiya, Anshul Sood

**Affiliations:** 1 Department of Medicine, Jawaharlal Nehru Medical College, Datta Meghe Institute of Higher Education and Research, Wardha, IND; 2 Department of Radiodiagnosis, Jawaharlal Nehru Medical College, Datta Meghe Institute of Higher Education and Research, Wardha, IND

**Keywords:** graves’ disease, auto immune disease, thyroid storm, thyrotoxic crisis, myasthenic crisis

## Abstract

When it comes to thyroid disorders, Graves' disease (GD) is the most prevalent autoimmune thyroid disorder in which antibodies are formed against thyroid receptors. Myasthenia gravis (MG) is a rare autoimmune neuromuscular junction disorder. Autoimmune antibodies are formed against postsynaptic neuromuscular junctions in MG, interrupting neuromuscular transmission and causing variable muscle weakness and tiredness. It affects older men and young women. The two disorders may coexist in a patient, or either of them may develop first. The thyroid condition GD is most frequently linked to MG. This case report describes an older man who presented with an acute exacerbation of MG along with a thyrotoxic crisis.

## Introduction

A thyrotoxic crisis is an acute, life-threatening, hypermetabolic state induced by the excessive release of thyroid hormone in individuals. Myasthenic crisis is a complication of myasthenia gravis (MG), which is characterized by worsening muscle weakness, resulting in respiratory failure that may require intubation and mechanical ventilation. These conditions are autoimmune in nature in which immune responses are directed toward self-component structures. Both of these illnesses share common characteristics, such as pathophysiological mechanisms and hereditary predisposition, but they also differ in specific ways, such as in their approach to treatment [1].

MG affects the postsynaptic neuromuscular junction. It is characterized by the production of antibodies mostly against postsynaptic cholinergic receptors and, to a lesser extent, against the protein of the muscle-specific kinase. This leads to variable muscle weakness. The majority of patients initially exhibit ptosis and diplopia before quickly progressing to widespread illness. Patients can experience acute respiratory failure as a result of this, and one distinctive aspect of this respiratory failure is that the neurologic process mediates it [2].

Thyrotoxicosis results from the production of autoantibodies against thyroid-stimulating hormone receptors in Graves’ disease (GD). Both these conditions are autoimmune disorders, and many individuals have reported having both conditions concurrently. Due to its mild signs, MG diagnosis is occasionally overlooked in ordinary clinical practice [3]. Physicians find it challenging to distinguish between ocular manifestations due to their similarity; as a result, extreme caution is required when doing so. By having immunomodulatory effects, hyperthyroid medication may worsen MG. Patients with MG experience worsening weakness due to beta-blockers and corticosteroids [4].

## Case presentation

A 54-year-old male, a shopkeeper by occupation, presented to the emergency department with complaints of difficulty in breathing for one day, with generalized weakness which increased as the day passed for 15 days. There was no history of fever, abdominal pain, chest pain, palpitations, loss of consciousness, or seizures. There was no history of hypertension, diabetes mellitus, bronchial asthma, or tuberculosis. He was a non-smoker and non-alcoholic.

**Figure 1:**
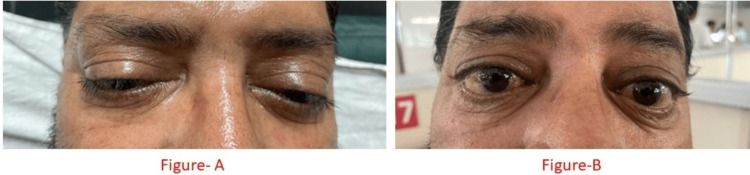
Showing on presentation, the patient had severe ptosis (A), which resolved after treatment (B)

On examination, the patient's vitals were as follows: pulse rate 124 beats/min, with a blood pressure of 140/90 mmHg. The patient had respiratory distress with a respiratory rate of 35 breaths/min and saturation of 92% on room air. The patient on presentation had ptosis (Figure 1). The central nervous system, abdomen, cardiovascular system, and respiratory system examinations were normal on systemic examination.

All routine investigations were done, and reports of the laboratory parameters are shown in Table 1.

**Table 1:** Routine laboratory parameters of the patient

Investigation	Patient’s value	Normal value
Haemoglobin	14 gm%	13-17 gm%
Total leucocyte count	12,000 cell/cumm	4000-10000 cells/cumm
Platelet	2,28,000 lacks/cumm	1.5-4.10 lacks/cumm
Mean corpuscular volume	80.6 fl	83-101 fl
Erythrocyte sedimentation rate	10 mm/1^st^ hr	1-10 mm/1^st^ hr
Free triiodothyronine (FT3)	3.21 pg/ml	2.77-5.27pg/ml
Free thyroxine (FT4)	1.78 ng/dl	0.78-2.19 ng/dl
Thyroid-stimulating hormone	0.076 mc IU/ml	0.465-4.68 mc IU/ml
Serum urea	40 mg/dl	19-43 mg/dl
Serum creatinine	1.1 mg/dl	0.6-1.25 mg/dl
Serum sodium	142 mmol/ml	137-145 mmol/ml
Serum potassium	3.8 mmol/ml	3.5-5.1 mmol/ml
Alkaline phosphatase	72 U/L	38-126 U/L
Alanine aminotransferase	108 U/L	17-59 U/L
Aspartate aminotransferase	59 U/L	17-59 U/L
Total protein/albumin	6.3/3.5 g/dl	6.3-8.2/3.5-5.0
Total bilirubin	1.4 mg/dl	0.2-1.3 mg/dl
Anti-thyroxine peroxidase antibody	54 IU/ml	0-34 IU/ml
Acetylcholine receptor autoantibody	>309	<0.45
Urine routine microscopy	Normal	Normal
Thyroid-stimulating hormone receptor autoantibody	5 IU/L	0-1 IU/L

**Figure 2:**
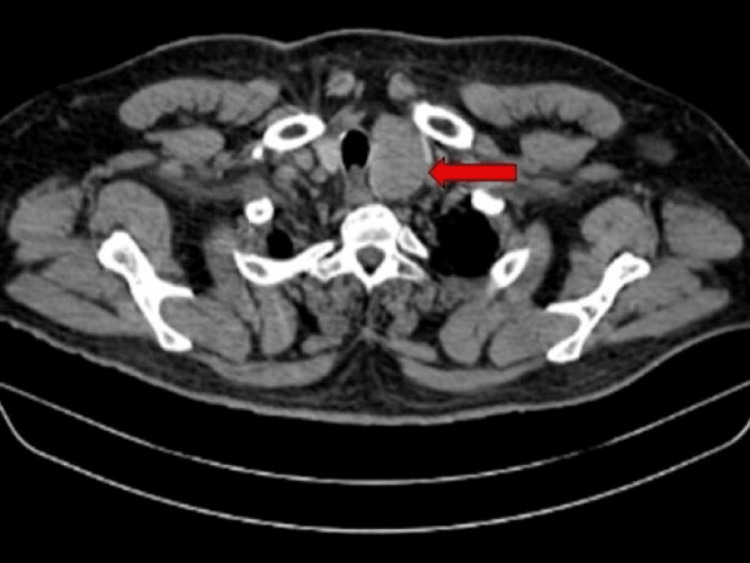
HRCT of the thorax showing a well-defined lesion of size 4.5 x 3.4 x 3.1 cm in the left lobe of the thyroid (red arrow)

During the hospital course, the patient's biochemical tests were sent, which revealed positive acetylcholine receptor autoantibody along with positive anti-TPO antibody and anti-TSH receptor antibody suggestive of the autoimmune nature of the disease. His high-resolution computed tomography (HRCT) of the thorax was done, which revealed normal lung parenchyma with a well-defined lesion of size 4.5 x 3.4 x 3.1 cm in the left lobe of the thyroid (Figure 2).

**Figure 3:**
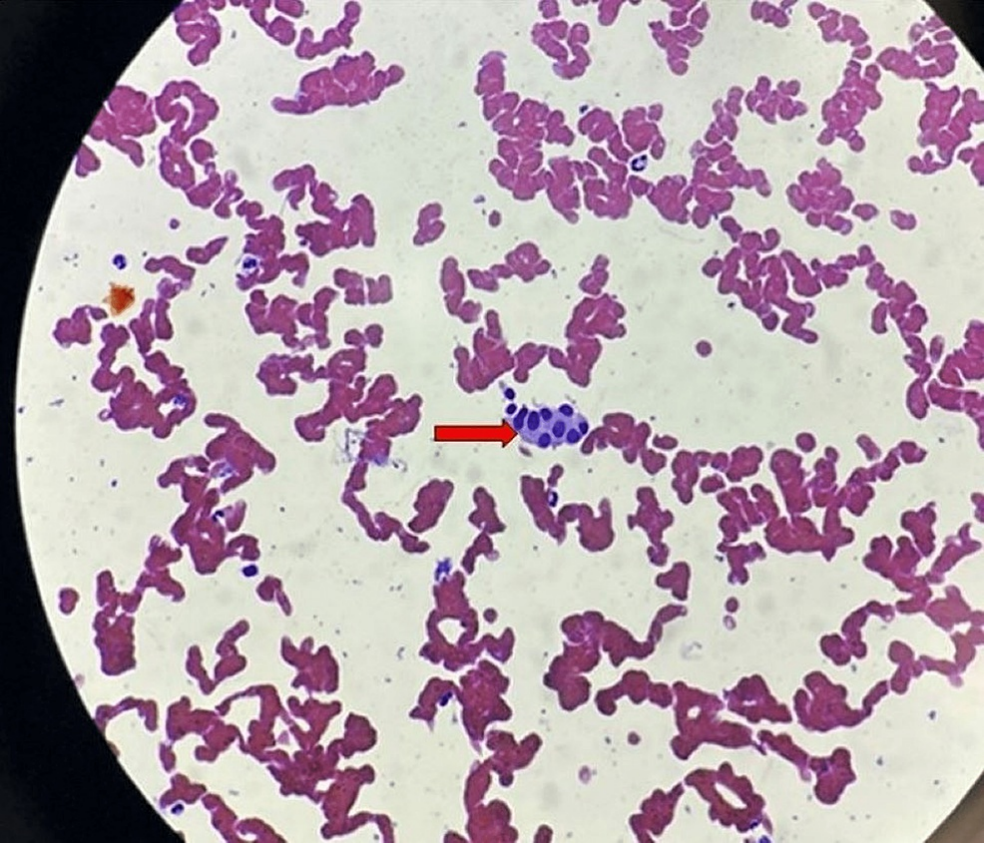
Cytology of FNAC of the thyroid gland shows follicular cells with a few hurthle cells (red arrow)

The patient's ultrasonography-guided fine needle aspiration cytology (FNAC) was done from the left lobe of the thyroid, and its cytology showed follicular cells with moderate to abundant pale cytoplasm and mild anisonucleosis, with occasional hurthle cells and a few cyst macrophages were seen. Cytology features suggested "hyperplastic follicular nodule" (Figure 3).

The patient was treated with intravenous hydrocortisone 100 mg thrice a day for 10 days, followed by oral steroids and acetylcholine esterase inhibitor pyridostigmine 300 mg daily in divided doses. The patient was also administered plasmapheresis for five cycles, after which the patient significantly improved symptomatically. The patient was started on tab Neomercazole with a dose of 20 mg thrice daily for newly diagnosed hyperthyroidism. During the hospital course, the patient was kept on non-invasive ventilation in view of tachypnoea. The patient was also given a trial of a beta-blocker (metoprolol) for sinus tachycardia, following which his tachypnoea worsened; hence, the beta blocker was withheld. In due course of time, the patient's symptoms improved. The ptosis resolved, and the need for non-invasive ventilation and further oxygen requirement of the patient decreased. The patient was discharged on tab pyridostigmine 120 mg twice daily, with tab Neomercazole 20 mg thrice daily. He was advised to follow up after 21 days. The patient was doing well on follow-up after one month.

## Discussion

The well-known autoimmune condition MG and hyperthyroidism GD frequently coexist in the same person [5]. Women are more likely to have MG than males [6]. Clinical symptoms of MG include fatigue and varied muscle weakness in different body muscle groups. Both diseases have comparable clinical characteristics, such as muscle weakening, in addition to sharing a similar pathophysiological pathway. It, thus, results in diagnostic uncertainty in individuals with both illnesses [7].

The skeletal muscles of the proximal limbs, the bulbar muscles, and the ocular muscles are among the many muscle groups in the body that exhibit varied muscle weakness and fatigue. Another paraneoplastic symptom of myasthenia is a thymus tumor. Even though our understanding of the pathophysiology and available treatments have improved, MG exacerbations occur more frequently in the first year after a diagnosis. The morbidity rates still remain high [8].

Thymus involvement in MG pathology includes thymus hyperplasia and thymomas, which are seen in early-onset and late-onset MG, respectively. Thymus pathology, such as thymomas and thymus hyperplasia, are also seen in GD. There is a case report of GD that exhibits symptoms similar to myasthenia and thymus enlargement on imaging, but diagnostic testing for MG was negative. Treating GD in that patient resulted in complete remission of symptoms and thymus enlargement [9]. Due to the difficulty in making a diagnosis, there are several methods for diagnosing MG with and without thyroid disease, and treating one pathology may make the other worse. In our patient, there was no evidence of thymoma, and he was diagnosed on the basis of the biochemical parameter, which is the acetylcholine receptor antibody.

Treatment for MG predominantly focuses on removing the auto-antibodies and complement inhibition as it is an antibody-mediated condition that causes B cell activation and, thus, complement activation [10]. Several methods, like plasmapheresis and complement inhibitor drugs like eculizumab, can help us achieve this goal. MG's recent interventions, like plasma exchange, double filtration plasmapheresis, and immunoadsorption, have decreased fatality rates with time.

The frequency of MG is higher in the elderly and is more common in women. Our patient was a 54-year-old adult male. The sensitivity and specificity of various diagnostic methods for MG, including the cold pack test, neostigmine test, electrophysiological studies, rest test, and circulating antibody testing, can vary. Hence, a positive biochemical test confirms the diagnosis [11].

Our patient was treated with intravenous steroids, whose dose was tapered off slowly over days. The patient was also administered plasmapheresis, to which the patient responded well, along with thionamides and choline esterase inhibitors as definitive therapy. Along with all the definitive treatment, the patient was given conservative management. He responded well to treatment and was discharged on choline esterase inhibitor and thionamide.

## Conclusions

Clinical similarities make it challenging to distinguish between GD and MG. There is a see-saw association between GD and MG. Therefore, clinicians should always consider MG as a differential in patients who present with new symptoms of exhaustion, respiratory failure, or neuromuscular weakness, such as hyperthyroidism, because treating one illness may worsen the other. There are still more relapses and myasthenia crises despite the usual myasthenia gravis treatment, which includes steroids, acetylcholinesterase inhibitors, rituximab, immunosuppressant, and thymectomy. The two novel therapies for MG, eculizumab and plasmapheresis, both show signs of significant improvement in recent studies.

Thus, a proper examination and thorough investigations can help diagnose both the conditions at the right time and the possible double crisis or, in other words, a double storm.
